# Wheat Chromatin Remodeling Protein TaSWP73 Contributes to Compatible Wheat–Powdery Mildew Interaction

**DOI:** 10.3390/ijms26062590

**Published:** 2025-03-13

**Authors:** Yixian Fu, Zige Yang, Jiao Liu, Xiaoyu Wang, Haoyu Li, Pengfei Zhi, Cheng Chang

**Affiliations:** College of Life Sciences, Qingdao University, Qingdao 266071, China

**Keywords:** wheat, chromatin remodeling protein, post-penetration resistance, nucleosome occupancy, *Blumeria graminis forma specialis tritici*

## Abstract

Wheat powdery mildew disease caused by the obligate biotrophic fungal pathogen *Blumeria graminis forma specialis tritici* (*B.g. tritici*) seriously threatens global wheat production. Although improved powdery mildew resistance is an aim in wheat breeding, the regulatory mechanism underlying the wheat–*B.g. tritici* interaction remains poorly understood. In this study, the wheat chromatin remodeling protein TaSWP73 was identified as a negative regulator of post-penetration resistance against *B.g. tritici.* The transient overexpression of *TaSWP73* attenuates wheat post-penetration resistance against *B.g. tritici*, while the silencing of *TaSWP73* potentiates salicylic acid (SA) biosynthesis and activates post-penetration resistance against *B.g. tritici.* Importantly, chromatin in the promoter regions of *TaSARD1,* an activator gene of SA biosynthesis, is marked by high nucleosome occupancy in the *TaSWP73*-silenced wheat leaves. The silencing of *TaSARD1* could suppress SA biosynthesis and attenuate post-penetration resistance against *B.g. tritici* with a lack of *TaSWP73.* In addition, TaICS1 was characterized as an essential component of wheat SA biosynthetic machinery. Potentiated SA biosynthesis and increased post-penetration resistance against *B.g. tritici* with a lack of *TaSWP73* could be suppressed by the silencing of *TaICS1* expression. These results collectively support the hypothesis that the wheat chromatin remodeling protein TaSWP73 contributes to the compatible wheat–powdery mildew interaction presumably via the suppression of the TaSARD1-TaICS1-SA pathway.

## 1. Introduction

As one of the most widely cultivated staple crops, allohexaploid bread wheat (*Triticum aestivum* L.) provides about one-fifth of the calories in human food [1,2]. The increasing global population drives the demand for wheat grains, but wheat growth and yields are threatened by host-adapted pathogens and pests (P&Ps) [3]. The biotrophic fungal pathogen *Blumeria graminis forma specialis tritici* (*B.g. tritici*) is the causal agent of the devastating powdery mildew disease, which results in a wheat yield loss of 10–40% [4,5]. During the infection, *B.g. tritici* conidia landing on the wheat host epidermal cells firstly germinate and then produce appressorium, enabling penetration through the plant cell wall [4,5]. In the post-penetration stages, a feeding structure of *B.g. tritici*, the haustorium, is developed to absorb nutrients from wheat cells, and finally, microcolonies are formed to disperse conidia [4,5]. Cultivating *B.g. tritici*-resistant wheat varieties is one of the most effective and economical ways to control this epidemic [4,5]. To this end, it is vital to decipher the regulatory mechanism underlying the wheat–*B.g. tritici* interaction.

The recognition of invading pathogens such as *B.g. tritici* by host plants like bread wheat could trigger induced defenses like pattern-triggered immunity (PTI) and effector-triggered immunity (ETI) to cope with pathogen infections [6,7,8,9,10,11,12,13,14,15,16]. Defense-related phytohormone salicylic acid (SA) plays a key role in initiating the intertwined PTI and ETI, and the activation of PTI and ETI usually culminates in massive transcriptomic reprogramming [17,18,19]. In the dicot model plant *Arabidopsis thaliana*, the major route for SA biosynthesis takes place in the chloroplast, and isochorismate synthase AtICS1 (isochorismate synthase 1) is one of the key enzymes in the SA biosynthetic machinery [20]. *Arabidopsis* calmodulin-binding protein SYSTEMIC ACQUIRED RESISTANCE DEFICIENT 1 (AtSARD1) was identified as a key regulator of *AtICS1* induction and SA biosynthesis [21]. In addition, epigenetic modulators like histone acetylases and chromatin remodeling proteins were characterized as key regulators of plant defense in *A. thaliana* [22]. For instance, the expression of *AtSARD1* and SA biosynthesis was epigenetically suppressed by histone deacetylase AtHDA6 [23]. In addition, *Arabidopsis* switch/sucrose non-fermentable (SWI/SNF)-associated protein AtSWP73A epigenetically suppresses the plant immune receptor [24]. However, the potential regulation of the wheat–*B.g. tritici* interaction by the chromatin remodeling protein SWP73 remains unknown.

Herein, the wheat chromatin remodeling protein TaSWP73 was identified as an epigenetic suppressor of post-penetration resistance against *B.g. tritici.* The transient overexpression of *TaSWP73* attenuates wheat post-penetration resistance against *B.g. tritici*, while the silencing of *TaSWP73* potentiates SA accumulation and activates post-penetration resistance against *B.g. tritici.* Importantly, chromatin in the promoter regions of *TaSARD1* is marked by high nucleosome occupancy in *TaSWP73*-silenced wheat leaves, suggesting that the chromatin remodeling protein TaSWP73 suppresses *TaSARD1* transcription at the epigenetic level. The silencing of *TaSARD1* and isochorismate synthase gene *TaICS1* could suppress SA biosynthesis and attenuate post-penetration resistance against *B.g. tritici* with a lack of *TaSWP73.* These results collectively support the hypothesis that the wheat chromatin remodeling protein TaSWP73 contributes to the compatible wheat–powdery mildew interaction presumably via the epigenetic suppression of *TaSARD1* transcription and the attenuation of the TaSARD1-TaICS1-SA pathway. These findings shed novel light on the epigenetic mechanism underlying wheat–*B.g. tritici* interactions and provide valuable information for genetic improvement in wheat resistance against devastating powdery mildew disease.

## 2. Results

### 2.1. Characterization of TaSWP73 Genes in Regulation of Compatible Wheat–B.g. tritici Interaction

**Figure 1:**
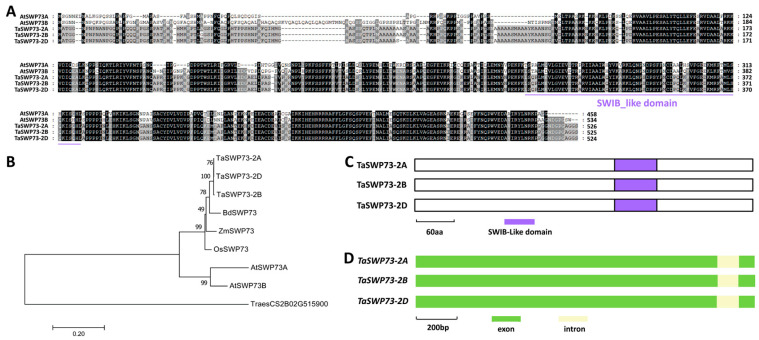
Identification of wheat TaSWP73 based on homology with *Arabidopsis* AtSWP73A and AtSWP73B. (**A**) Protein sequence alignments of *Arabidopsis* AtSWP73A, AtSWP73B, wheat TaSWP73-2A, TaSWP73-2B, and TaSWP73-2D. Conserved residues among 5 protein sequences are shaded in dark, while residues conserved in at least 3 of the 5 proteins are shaded in gray. (**B**) Phylogenetic relationships of the SWP73 proteins from *Arabidopsis*, *Brachypodium*, maize, rice, and wheat. Two-letter genus species prefixes: *At*, *Arabidopsis thaliana*; *Bd*, *Brachypodium distachyon*; *Os*, *Oryza sativa*; *Ta*, *Triticum aestivum*; *Zm*, *Zea mays*. (**C**) Domain structures of wheat TaSWP73-2A, TaSWP73-2B, and TaSWP73-2D proteins. (**D**) Gene architectures of wheat *TaSWP73-2A*, *TaSWP73-2B*, and *TaSWP73-2D* genes.

In this study, we are interested in examining the function of wheat SWP73 homologs in the wheat–*B.g. tritici* interaction. To this end, amino acid sequences of *Arabidopsis* AtSWP73A (At3g01890) and AtSWP73B (At5g14170) were used as queries to search the reference genome of the hexaploid bread wheat (http://plants.ensembl.org/). TaSWP73 was identified as the wheat homolog of AtSWP73A and AtSWP73B. Three *TaSWP73* genes separately located on wheat chromosomes 2A, 2B, and 2D were designated as *TaSWP73-2A* (*TraesCS2A02G281500*), *TaSWP73-2B* (*TraesCS2B02G298800*), and *TaSWP73-2D* (*TraesCS2D02G280300*) (Figure S1). As shown in Figure 1A, these predicted TaSWP73-2A, TaSWP73-2B, and TaSWP73-2D proteins shared more than 57% of their identity with *Arabidopsis* AtSWP73A and AtSWP73B. Phylogenetic analysis validated that wheat TaSWP73-2A, TaSWP73-2B, and TaSWP73-2D proteins are homologs of *Brachypodium* BdSWP73, maize ZmSWP73, rice OsSWP73, and *Arabidopsis* TaSWP73A and TaSWP73B (Figure 1B). As shown in Figure 1C,D, a conserved SWIB-like domain was identified in all TaSWP73 proteins, and the coding regions of *TaSWP73* genomic sequences all contained two exons and one intron.

**Figure 2:**
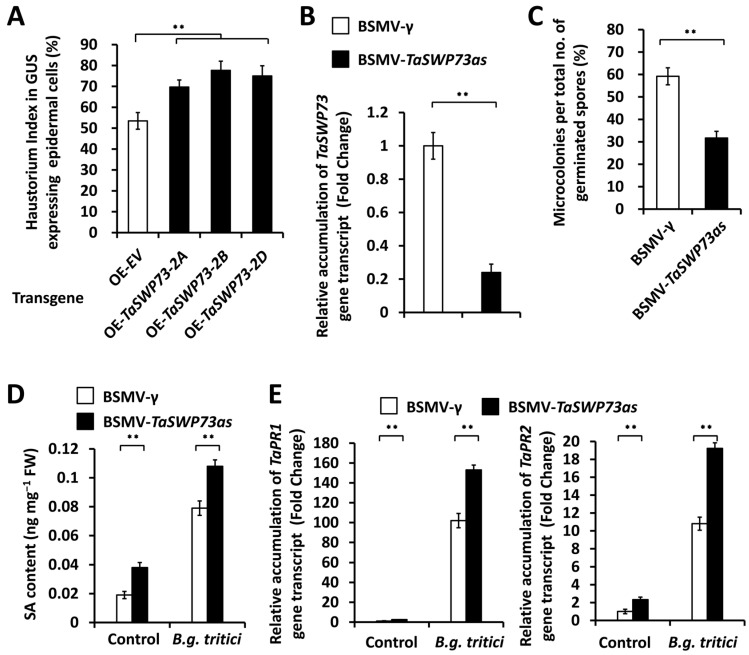
Functional characterization of *TaSWP73* gene in wheat–*B.g. tritici* interaction. (**A**) Statistical analysis of *B.g. tritici* haustorial formation in wheat epidermal cells transiently overexpressing *TaSWP73* (OE-*TaSWP73*). Wheat epidermal cells bombarded with empty vector (OE-EV) were set as a control. More than 50 wheat cells were analyzed for each experiment. (**B**) RT-qPCR analysis of *TaSWP73* transcript accumulation in the wheat leaves silencing *TaSWP73* (BSMV-*TaSWP73as*). (**C**) Statistical analysis of *B.g. tritici* microcolony formation in wheat leaves silencing *TaSWP73*. (**D**) Measurement of SA content in wheat leaves silencing *TaSWP73*. (**E**) RT-qPCR analysis of *TaPR1* and *TaPR2* transcript accumulation in wheat leaves silencing *TaSWP73*. For (**B**–**E**), leaves of wheat plants infected with BSMV-γ were employed as the negative control. For (**A**–**E**), three technical replicates per treatment were statistically analyzed, and data are presented as the mean ± SE (Student’s *t*-test; ** *p* < 0.01), and these assays were repeated in three independent biological replicates with similar results.

To analyze the function of the *TaSWP73* gene in the regulation of the wheat–*B.g. tritici* interaction, transient gene expression assays were performed to overexpress the *TaSWP73-2A*, *TaSWP73-2B*, and *TaSWP73-2D* genes in the wheat leaf epidermal cells. These bombarded wheat leaves were inoculated with *B.g. tritici* conidia, and the *B.g. tritici* haustorium index (HI%) was analyzed. As shown in Figure 2A, the *B.g. tritici* HI% increased from 54.6% for the empty vector (OE-EV) control to above 69.7% for wheat cells overexpressing the *TaSWP73-2A*, *TaSWP73-2B*, or *TaSWP73-2D* gene, suggesting that the *TaSWP73* gene negatively regulates wheat post-penetration resistance against powdery mildew and positively contributes to the *B.g. tritici* post-penetration event haustorial formation. Thereafter, we employed barley stripe mosaic virus (BSMV)-induced gene silencing (BSMV-VIGS) to silence all endogenous *TaSWP73* genes in the wheat leaves. As shown in Figure 2B, the reverse transcription–quantitative polymerase chain reaction (RT-qPCR) assay demonstrated that the accumulation level of *TaSWP73* gene transcripts decreased significantly in wheat leaves silencing the *TaSWP73* gene. These BSMV-VIGS wheat leaves were inoculated with *B.g. tritici* conidia, and the *B.g. tritici* microcolony index (MI%) was analyzed. As shown in Figure 2C, the *B.g. tritici* MI% decreased from 59.3% for the control plants (BSMV-γ) to 31.7% for *TaSWP73*-silenced plants (BSMV-*TaSWP73as*), confirming that the *TaSWP73* gene negatively regulates wheat post-penetration resistance against *B.g. tritici* and positively contributes to the *B.g. tritici* post-penetration event microcolony formation. We measured the accumulation of defense-related phytohormone SA in *TaSWP73*-silenced wheat leaves infected by *B.g. tritici*. As shown in Figure 2D, the SA level was remarkably elevated in the *TaSWP73*-silenced wheat leaves, compared with that of the BSMV-γ control plants, suggesting that TaSWP73 suppresses SA accumulation in bread wheat. We analyzed the transcript levels of SA signaling marker genes *TaPR1* and *TaPR2* in *TaSWP73*-silenced wheat leaves. As shown in Figure 2E, the accumulation levels of the *TaPR1* and *TaPR2* transcripts were greatly enhanced by the silencing of *TaSWP73*. These results suggested that the chromatin remodeling protein TaSWP73 negatively regulates SA accumulation and contributes to the compatible wheat–powdery mildew interaction.

### 2.2. Epigenetic Regulation of TaSARD1 Genes by TaSWP73

**Figure 3:**
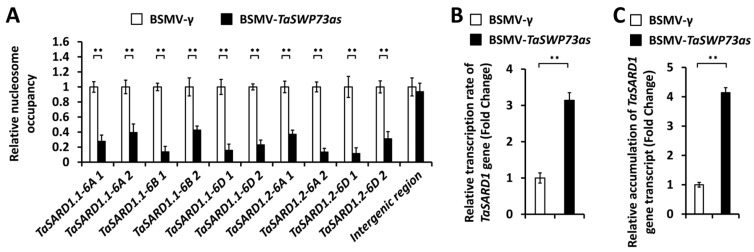
Characterization of nucleosomal occupancy and gene transcription at *TaSARD1* loci in *TaSWP73*-silenced wheat leaves. (**A**) MNase analysis of nucleosome occupancy in *TaSARD1* promoters in wheat leaves silencing *TaSWP73* (BSMV-*TaSWP73as*). The nucleosome occupancy levels in wheat leaves infected with the BSMV-γ empty vector (negative control) were set to 1.0. Transcription rates (**B**) and expression levels (**C**) of the *TaSARD1* gene in wheat leaves silencing *TaSWP73* were measured by nuclear run-on and RT-qPCR assays, respectively. For (**A**–**C**), leaves of wheat plants infected with BSMV-γ were employed as the negative control. For (**A**–**C**), three technical replicates per treatment were statistically analyzed, and data are presented as the mean ± SE (Student’s *t*-test; ** *p* < 0.01), and these assays were repeated in three independent biological replicates with similar results.

In the dicot model plant *A. thaliana*, the transcription of the *AtSARD1* gene is tightly regulated at epigenetic levels [23]. TaSARD1 genes were identified as key regulators of wheat post-penetration resistance against *B.g. tritici* [25]. We ask whether the wheat chromatin remodeling protein TaSWP73 is involved in the epigenetic regulation of the *TaSARD1* gene. To examine the potential regulation of the chromatin structure in promoter regions of *TaSARD1* genes by the wheat chromatin remodeling protein TaSWP73, the nucleosome occupancy micrococcal nuclease (MNase) assay was performed (Figure 3A). As shown in Figure 3A, the MNase assay showed significantly reduced nucleosome occupancy in *TaSARD1* promoters in the wheat leaves silencing the *TaSWP73* gene compared with the BSMV-γ control, suggesting that the wheat chromatin remodeling protein *TaSWP73* might function as an epigenetic repressor of the *TaSARD1* gene. Consistent with this, nuclear run-on and RT-qPCR assays demonstrated that the silencing of the *TaSWP73* gene resulted in a significant enhancement in the transcription rates and transcript accumulation of the *TaSARD1* gene (Figure 3B,C). These results suggested that the chromatin remodeling protein TaSWP73 contributes to the establishment of a repressive chromatin state in the *TaSARD1* gene.

### 2.3. Functional Analysis of TaSARD1 Genes in the TaSWP73-Mediated Suppression of SA Accumulation and Compatible Wheat–B.g. tritici Interaction

**Figure 4:**
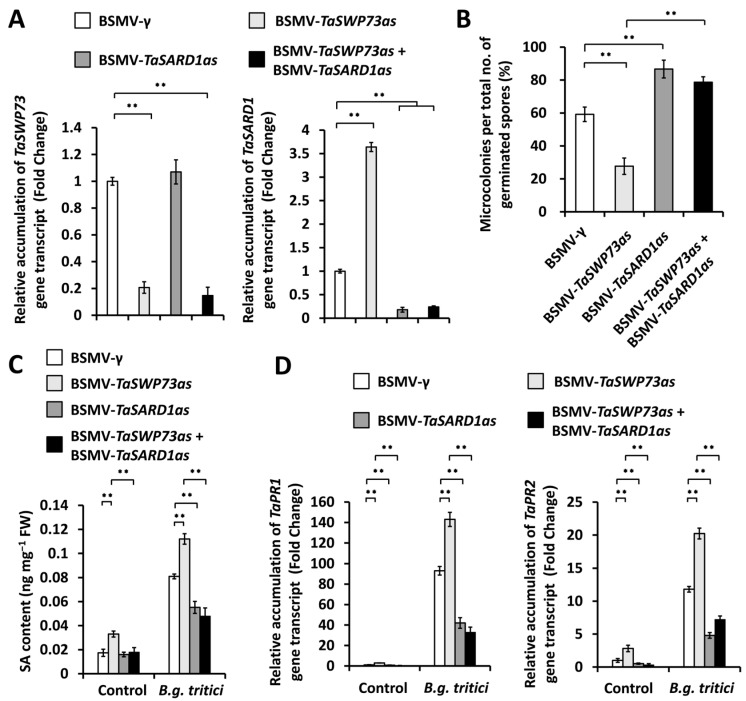
Characterization of the genetic interplay between *TaSWP73* and *TaSARD1* in the wheat–*B.g. tritici* interaction. (**A**) RT-qPCR analysis of *TaSWP73* and *TaSARD1* transcript accumulation in wheat leaves silencing *TaSWP73* (BSMV-*TaSWP73as*) or *TaSARD1* (BSMV-*TaSARD1as*) or co-silencing *TaSWP73* and *TaSARD1* (BSMV-*TaSWP73as* + BSMV-*TaSARD1as*). (**B**) Statistical analysis of *B.g. tritici* microcolony formation in wheat leaves silencing *TaSWP73* or *TaSARD1* or co-silencing *TaSWP73* and *TaSARD1*. (**C**) Measurement of SA content in wheat leaves silencing *TaSWP73* or *TaSARD1* or co-silencing *TaSWP73* and *TaSARD1*. (**D**) RT-qPCR analysis of *TaPR1* and *TaPR2* transcript accumulation in wheat leaves silencing *TaSWP73* or *TaSARD1* or co-silencing *TaSWP73* and *TaSARD1*. For (**A**–**D**), leaves of wheat plants infected with BSMV-γ were employed as the negative control. For (**A**–**D**), three technical replicates per treatment were statistically analyzed, and data are presented as the mean ± SE (Student’s *t*-test; ** *p* < 0.01), and these assays were repeated in three independent biological replicates with similar results.

Having already demonstrated that *TaSARD1* gene expression and wheat post-penetration resistance against *B.g. tritici* are negatively regulated by the chromatin remodeling protein *TaSWP73*, we next ask whether the chromatin remodeling protein *TaSWP73* negatively regulates wheat post-penetration resistance against *B.g. tritici* via the suppression of *TaSARD1* gene expression. To test this hypothesis, we simultaneously silenced the *TaSWP73* and *TaSARD1* genes and analyzed the *B.g. tritici* MI%. As shown in Figure 4A, the accumulation levels of the *TaSWP73* or *TaSARD1* gene transcript decreased remarkably in wheat leaves co-silencing *TaSWP73* and *TaSARD1* genes, compared with the BSMV-γ control. The *B.g. tritici* MI% decreased from 58.9% for the control plants (BSMV-γ) to 30.2% for the *TaSWP73*-silenced (BSMV-*TaSWP73as*) plants but increased to above 77.2% for wheat leaves co-silencing *TaSWP73* with the *TaSARD1* gene (Figure 4B). Under *B.g. tritici* infection, the SA level showed a significant increase in the wheat leaves silencing the *TaSWP73* gene but a remarkable reduction in the wheat leaves co-silencing *TaSWP73* with the *TaSARD1* gene (Figure 4C). The RT-qPCR assay further demonstrated that the accumulation levels of *TaPR1* and *TaPR2* transcripts significantly increased in the wheat leaves silencing the *TaSWP73* gene but remarkably decreased in the wheat leaves co-silencing the *TaSWP73* and *TaSARD1* genes, compared with the BSMV-γ control (Figure 4D). The above results indicated that potentiated SA biosynthesis and increased post-penetration resistance against *B.g. tritici* with a lack of *TaSWP73* could be attenuated by the silencing of *TaSARD1* expression, suggesting that the epigenetic suppression of *TaSARD1* by the chromatin remodeling protein *TaSWP73* might contribute to the negative regulation of *TaSWP73* in SA biosynthesis and a positive contribution to the compatible wheat–powdery mildew interaction.

### 2.4. Functional Analysis of TaICS1 Genes in the TaSWP73-Mediated Suppression of SA Accumulation and Wheat–B.g. tritici Interaction

**Figure 5:**
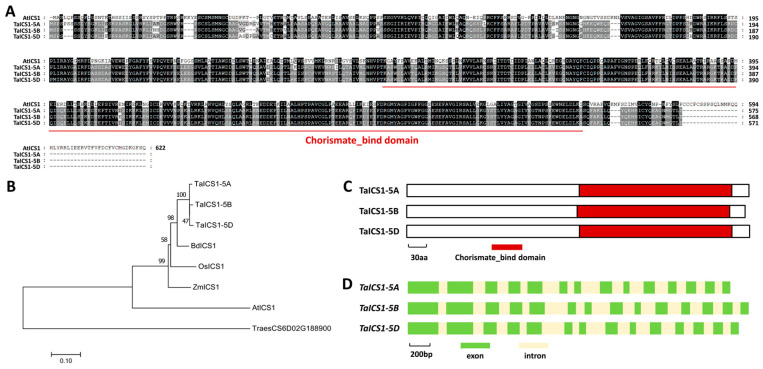
Identification of wheat TaICS1 based on homology with *Arabidopsis* AtICS1. (**A**) Protein sequence alignments of *Arabidopsis* AtICS1, wheat TaICS1-5A, TaICS1-5B, and TaICS1-5D. Conserved residues among 4 protein sequences are shaded in dark, while residues conserved in at least 2 of the 4 proteins are shaded in gray. (**B**) Phylogenetic relationships of the ICS1 proteins from *Arabidopsis*, *Brachypodium*, maize, rice, and wheat. Two-letter genus species prefixes: *At*, *Arabidopsis thaliana*; *Bd*, *Brachypodium distachyon*; *Os*, *Oryza sativa*; *Ta*, *Triticum aestivum*; *Zm*, *Zea mays*. (**C**) Domain structures of wheat TaICS1-5A, TaICS1-5B, and TaICS1-5D proteins. (**D**) Gene architectures of wheat *TaICS1-5A*, *TaICS1-5B*, and *TaICS1-5D* genes.

In the dicot model plant *A. thaliana*, AtICS1 is involved in SA biosynthesis [20]. Herein, we are interested in examining the function of wheat ICS1 homologs in the wheat–*B.g. tritici* interaction. To this end, we first searched the reference genome of the hexaploid bread wheat using the amino acid sequence of *Arabidopsis* AtICS1 (At1g74710) as a query and identified TaICS1 as a wheat homolog of AtICS1. Three *TaICS1* genes separately located on wheat chromosomes 5A, 5B, and 5D were designated as *TaICS1-5A* (*TraesCS5A02G193800*), *TaICS1-5B* (*TraesCS5B02G189100*), and *TaICS1-5D* (*TraesCS5D02G196200*). As shown in Figure 5A, these predicted TaICS1-5A, TaICS1-5B, and TaICS1-5D proteins shared more than 48% of their identity with *Arabidopsis* AtICS1. Phylogenetic analysis validated that wheat TaICS1-5A, TaICS1-5B, and TaICS1-5D proteins are homologs of *Brachypodium* BdICS1, maize ZmICS1, rice OsICS1, and *Arabidopsis* TaICS1 (Figure 5B). As shown in Figure 5C,D, a conserved chorismate-binding enzyme (Chorismate_bind) domain was identified in the C-terminal parts of all TaICS1 proteins, and the coding regions of the *TaICS1* genomic sequences all contained 14 exons and 13 introns.

**Figure 6:**
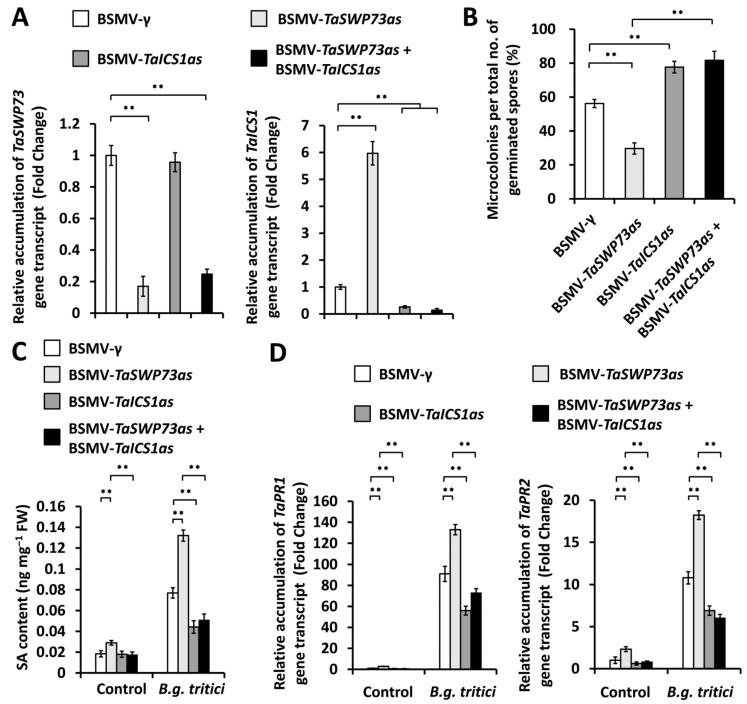
Characterization of the genetic interplay between *TaSWP73* and *TaICS1* in the wheat–*B.g. tritici* interaction. (**A**) RT-qPCR analysis of *TaSWP73* and *TaICS1* transcript accumulation in wheat leaves silencing *TaSWP73* (BSMV-*TaSWP73as*) or *TaICS1* (BSMV-*TaICS1as*) or co-silencing *TaSWP73* and *TaICS1* (BSMV-*TaSWP73as* + BSMV-*TaICS1as*). (**B**) Statistical analysis of *B.g. tritici* microcolony formation in wheat leaves silencing *TaSWP73* or *TaICS1* or co-silencing *TaSWP73* and *TaICS1*. (**C**) Measurement of SA content in wheat leaves silencing *TaSWP73* or *TaICS1* or co-silencing *TaSWP73* and *TaICS1*. (**D**) RT-qPCR analysis of *TaPR1* and *TaPR2* transcript accumulation in wheat leaves silencing TaSWP73 or *TaICS1* or co-silencing *TaSWP73* and *TaICS1*. For (**A**–**D**), leaves of wheat plants infected with BSMV-γ were employed as the negative control. For (**A**–**D**), three technical replicates per treatment were statistically analyzed, and data are presented as the mean ± SE (Student’s *t*-test; ** *p* < 0.01), and these assays were repeated in three independent biological replicates with similar results.

To examine the function of the *TaICS1* genes in the *TaSWP73*-mediated suppression of SA accumulation and wheat post-penetration resistance against *B.g. tritici*, we simultaneously silenced *TaSWP73* and *TaICS1* genes and analyzed the *B.g. tritici* MI%. As shown in Figure 6A, the accumulation levels of the *TaSWP73* or *TaICS1* gene transcripts decreased remarkably in wheat leaves co-silencing *TaSWP73* and *TaICS1* genes, compared with the BSMV-γ control. The *B.g. tritici* MI% decreased from 56.8% for the control plants (BSMV-γ) to 29.7% for *TaSWP73*-silenced (BSMV-*TaSWP73as*) leaves but increased to above 76.9% for wheat leaves co-silencing *TaSWP73* with the *TaICS1* gene (Figure 6B). Under *B.g. tritici* infection, the SA level showed a significant increase in the wheat leaves silencing the *TaSWP73* gene but a remarkable reduction in the wheat leaves co-silencing *TaSWP73* with the *TaICS1* gene (Figure 6C). The RT-qPCR assay further demonstrated that the accumulation levels of *TaPR1* and *TaPR2* gene transcripts significantly increased in the wheat leaves silencing the *TaSWP73* gene but remarkably decreased in the wheat leaves co-silencing *TaSWP73* with the *TaICS1* gene, compared with the BSMV-*γ* control (Figure 6D). The above results indicated that potentiated SA biosynthesis and increased post-penetration resistance against *B.g. tritici* with a lack of *TaSWP73* could be attenuated by the silencing of *TaICS1* expression. These findings collectively suggested that the wheat chromatin remodeling protein TaSWP73 contributes to the compatible wheat–powdery mildew interaction probably via the suppression of the *TaSARD1*-*TaICS1*-SA pathway.

## 3. Discussion

### 3.1. Wheat Chromatin Remodeling Protein TaSWP73 Positively Contributes to the Compatible Wheat–B.g. tritici Interaction

In this study, three *TaSWP73* genes (*TaSWP73-2A*, *TaSWP73-2B*, and *TaSWP73-2D*) were separately identified from wheat chromosomes 2A, 2B and 2D. Sequence alignment and phylogenetic analysis demonstrated that the highly homologous TaSWP73-2A, TaSWP73-2B, and TaSWP73-2D proteins are the closest homologs of *Brachypodium* BdSWP73, maize ZmSWP73, rice OsSWP73, and *Arabidopsis* TaSWP73A and TaSWP73B. The overexpression of *TaSWP73-2A*, *TaSWP73-2B*, and *TaSWP73-2D* resulted in a significantly increased *B.g. tritici* haustorium index (HI%), while silencing *TaSWP73* led to a remarkably decreased *B.g. tritici* microcolony index (MI%), suggesting that the wheat chromatin remodeling protein *TaSWP73* negatively regulates post-penetration resistance against powdery mildew and positively contributes to *B.g. tritici* post-penetration events like haustorium development and microcolony formation. Interestingly, SA accumulation and the expression levels of SA signaling-related defense marker genes *TaPR1* and *TaPR2* were potentiated in the *TaSWP73*-silenced wheat leaves, suggesting that TaSWP73 negatively regulates SA accumulation. Interestingly, AtSWP73A was revealed to suppress the *Arabidopsis* defense response against the bacterium *Pseudomonas syringae* pv. *tomato* (*Pst*) strain carrying the effector AvrRpt2 [24]. These studies imply that SWP73 homologs negatively regulate plant resistance against (hemi)biotrophic pathogenic bacteria and fungi in the dicot *Arabidopsis* and monocot bread wheat. Interestingly, RT-qPCR demonstrated that the accumulation level of *TaSWP73* gene transcripts was not significantly changed by *B.g. tritici* inoculation (Figure S2), suggesting that the suppression of wheat defense by TaSWP73 is maintained during *B.g. tritici* infection.

### 3.2. Isochorismate Synthase TaICS1 Positively Contributes to SA Biosynthesis and Wheat Post-Penetration Resistance Against B.g. tritici

Herein, three *TaICS1* genes (*TaICS1-5A*, *TaICS1-5B*, and *TaICS1-5D*) were separately identified from wheat chromosomes 5A, 5B, and 5D. Sequence alignment and phylogenetic analysis demonstrated that the highly homologous TaICS1-5A, TaICS1-5B, and TaICS1-5D proteins are the closest homologs of *Brachypodium* BdICS1, maize ZmICS1, rice OsICS1, and *Arabidopsis* AtICS1. The *B.g. tritici* haustorium index (HI%) and microcolony index (MI%) were enhanced by the silencing of the *TaICS1* gene, suggesting that wheat isochorismate synthase TaICS1 positively regulates post-penetration resistance against powdery mildew and suppresses *B.g. tritici* post-penetration events like haustorium development and microcolony formation. Notably, the knockdown of the *TaICS1* gene attenuated the SA accumulation in wheat leaves under *B.g. tritici* infection. In contrast, SA accumulation in the absence of *B.g. tritici* infection was not significantly affected by the silencing of the *TaICS1* gene, indicating that wheat isochorismate synthase TaICS1 specifically contributes to SA biosynthesis under *B.g. tritici* infection. Consistent with this study, Zhang et al. reported that the mutation of one or two *TaICS1* homoeoalleles in wheat reduced the SA levels under ultraviolet treatment and *Fusarium graminearum* infection, further enhancing susceptibility to Fusarium head blight (FHB) [26]. These studies suggested that wheat *TaICS1* mainly governs the endogenous SA levels under infection with pathogens like *B.g. tritici* and *F. graminearum.* Interestingly, isochorismate synthase OsICS1 was reported to be required for phylloquinone biosynthesis in rice, and it would be intriguing to examine the potential regulation of phylloquinone biosynthesis by *TaICS1* in future research [27].

### 3.3. Wheat Chromatin Remodeling Protein TaSWP73 Positively Contributes to the Compatible Wheat–Powdery Mildew Interaction Presumably via Suppression of the TaSARD1-TaICS1-SA Pathway

**Figure 7:**
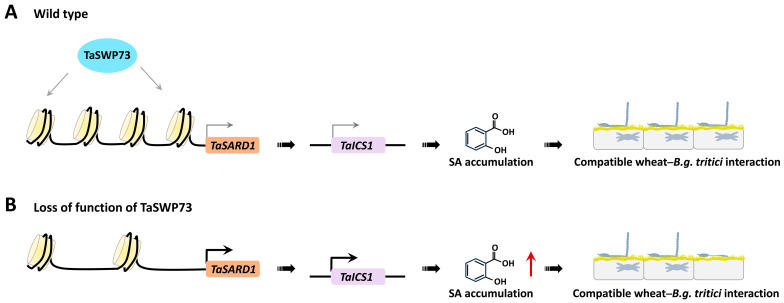
Proposed model for regulation of the compatible wheat–powdery mildew interaction by the chromatin remodeling protein TaSWP73. In the wild-type wheat plants (**A**), the wheat chromatin remodeling protein TaSWP73 facilitates chromatin assembly in promoter regions of the *TaSARD1* gene and maintains the *TaSARD1* gene transcription in the resting state. Other elusive chromatin remodeling protein might be involved in chromatin disassembly in the *TaSARD1* promoter to keep the basal expression of the *TaSARD1* gene. As a result, the expression level of the SA biosynthesis gene *TaICS1* is low, and SA accumulation is maintained at a basal level, leading to a fully compatible wheat–*B.g. tritici* interaction. In the absence of chromatin remodeling protein TaSWP73 (**B**), chromatin at *TaSARD1* promoters remains in an activated state marked by reduced nucleosomal occupancy, leading to the epigenetic activation of the *TaSARD1* gene. As a result, the expression of the SA biosynthesis gene *TaICS1* is up-regulated and SA accumulation is increased, leading to an attenuated compatible wheat–*B.g. tritici* interaction.

In this study, the MNase assay demonstrated that the wheat chromatin remodeling protein TaSWP73 facilitates chromatin assembly in promoter regions of the *TaSARD1* gene. The silencing of the *TaSWP73* gene resulted in reduced nucleosomal occupancy in *TaSARD1* promoters and potentiated *TaSARD1* transcription. The knockdown of *TaSARD1* and its downstream gene *TaICS*1 could attenuate SA biosynthesis and post-penetration resistance against *B.g. tritici* with a lack of *TaSWP73*, suggesting that the epigenetic suppression of *TaSARD1* by chromatin remodeling protein TaSWP73 might contribute to the negative regulation of SA biosynthesis and post-penetration resistance against *B.g. tritici* by *TaSWP73.* Based on these results, we propose a model of how the chromatin remodeling protein TaSWP73 functions in the regulation of the wheat–*B.g. tritici* interaction. In this model, depicted in Figure 7, the wheat chromatin remodeling protein TaSWP73 facilitates chromatin assembly in promoter regions of the *TaSARD1* gene and maintains the *TaSARD1* gene transcription in the resting state. Other elusive chromatin remodeling proteins might be involved in the chromatin disassembly in the *TaSARD1* promoter to keep the basal expression of the *TaSARD1* gene. As a result, the expression level of the SA biosynthesis gene *TaICS1* is low, and SA accumulation is maintained at a basal level, leading to a fully compatible wheat–*B.g. tritici* interaction. In the absence of the chromatin remodeling protein TaSWP73, chromatin in *TaSARD1* promoters remains in an activated state marked by reduced nucleosomal occupancy, leading to the epigenetic activation of the *TaSARD1* gene. As a result, the expression of the SA biosynthesis gene *TaICS1* is up-regulated and SA accumulation is increased, leading to an attenuated compatible wheat–*B.g. tritici* interaction.

Wheat histone deacetylase TaHDA6 and TaHDT701 were previously identified as epigenetic suppressors of post-penetration resistance against *B.g. tritici* [28,29]. As discussed by prior reviews, different types of epigenetic modulators like chromatin remodeling proteins and histone deacetylases could function in concert to regulate plant development and environmental adaptation [30]. Therefore, it is intriguing to examine the potential interplays between the chromatin remodeling protein TaSWP73 and histone deacetylases TaHDA6 and TaHDT701 in the epigenetic suppression of SA biosynthesis and post-penetration resistance against *B.g. tritici.* In addition, calmodulin-binding transcription activators (CAMTAs) TaCAMTA2 and TaCAMTA3 were previously identified as suppressors of wheat post-penetration resistance against powdery mildew [25]. Interestingly, the expressions of *TaSARD1* and *TaEDS1* were potentiated by the silencing of the *TaCAMTA2* and *TaCAMTA3* genes, suggesting that transcription factors TaCAMTA2 and TaCAMTA3 negatively regulate the expression of the *TaSARD1* and *TaEDS1* genes [25]. Analyzing the potential association of the chromatin remodeling protein TaSWP73 with transcription factors TaCAMTA2 and TaCAMTA3 might shed novel light on the molecular mechanism underlying TaSWP73 function in SA biosynthesis and the wheat–*B.g. tritici* interaction in future research.

Herein, the wheat *TaSWP73* gene was identified as a *susceptibility* (*S*) gene contributing to the establishment of a compatible wheat–*B.g. tritici* interaction [31,32,33]. As summarized by previous reviews, a plethora of *S* genes such as *TaMLO*, *TaEDR1*, *TaPOD70*, and *TaDND1/2* have been identified [34,35,36,37,38]. Inactivating the *S* genes *TaMLO* and *TaEDR1* via newly developed genome editing and targeting induced local lesions in genomes (TILLING) techniques could reduce wheat compatibility with *B.g. tritici* and confer durable resistance [39,40,41,42,43,44,45,46]. Therefore, genetically manipulating the *TaSWP73* gene by TILLING or genome editing approaches like transcription activator-like effector nucleases (TALENs) and CRISPR (clustered regularly interspaced short palindromic repeats)–Cas 9 (CRISPR-associated 9) might provide a new avenue for breeding new wheat varieties with improved powdery mildew resistance.

## 4. Materials and Methods

### 4.1. Plant and Fungal Materials

*B.g. tritici*-susceptible wheat cultivar Yannong 999 and virulent *B.g. tritici* isolate E09 were employed for wheat–*B.g. tritici* interaction characterization in this study. Wheat seedlings were grown in growth chambers under 16 h light/8 h dark with a light intensity of 150 μmol photons s^−1^m^−2^, a 20 °C/18 °C day/night cycle, and 70% relative humidity (RH). *B.g. tritici* isolate E09 was maintained on the plants of wheat cultivar Yannong 999 and kept at 70% RH and a 20 °C day/18 °C night cycle.

### 4.2. Gene Expression Analysis

Reverse transcription–quantitative polymerase chain reaction (RT-qPCR) and nuclear run-on assays were performed to analyze the transcript accumulation and transcription rates of the *TaSWP73*, *TaSARD1*, and *TaICS1* genes. The newly grown wheat leaves (n = 5, randomly chosen) with virus symptoms about two weeks post BSMV infection were harvested for the RT-qPCR and nuclear run-on assays. For the RT-qPCR assay, the total RNA was extracted from the wheat leaves using TRizol solution and treated with RNase-free DNase I to remove potential DNA contamination. The first-strand cDNA was synthesized using 1 μg of the total RNA and used in RT-qPCR as a template to detect the expression of the indicated wheat gene. The RT-qPCR assay was performed using the ABI step-one real-time PCR system with the GoTaq qPCR Master Mix. The expression of *TaEF1* was set as the internal control, and the expression levels of *TaSWP73*, *TaSARD1*, *TaICS1*, *TaPR1*, or *TaPR2* were measured by qPCR using the qPCR Master Mix (Invitrogen, Waltham, MA, USA) under the following programs: 95 °C for 3 min, 40 cycles at 95 °C for 20 s, 56 °C for 30 s, and 72 °C for 15 s, followed by 72 °C for 1 min. For the nuclear run-on assay, wheat cell nuclei were isolated and mixed with reaction buffer (25 mM biotin-16-UTP and 0.75 mM of ATP, CTP, and GTP) for the transcription reaction. After RNA extraction, the nascent RNA was enriched by streptavidin magnetic beads and subjected to the RT-qPCR assay. In the RT-qPCR and nuclear run-on assays, *TaEF1*, *TaSWP73*, *TaSARD1*, *TaICS1*, *TaPR1*, and *TaPR2* were analyzed using the primers 5′CAGGACGTTTACAAGATTG3′/5′CAAAACCACGCTTCAGATC3′, 5′CTTATAAGGCTGCTAACTC3′/5′GGGACGGTGGTGTCTTGAG3′, 5′GCGAGTAATGAAAGCAT3′/5′TTAATCAACTTGATCCC3′, 5′CCACAAGGAGCAGTGGGAG3′/5′TGTGGAACAACGAAGTAGA3′, 5′GAGAATGCAGACGCCCAAG3′/5′TGGAGCTTGCAGTCGTTGATC3′, and 5′AGGATGTTGCTTCCATGTTTG3′/5′AGTAGATGCGCATGCCGTTG3′. For the RT-qPCR and nuclear run-on assays, three technical replicates using replicate samples were statistically analyzed, and the data are presented as the mean ± SE (Student’s *t*-test; ** *p* < 0.01). All RT-qPCR and nuclear run-on assays were repeated in three biological replicates using independently prepared samples with similar results.

### 4.3. BSMV-Mediated Gene Silencing and B.g. tritici Microcolony Formation Analysis

Barley stripe mosaic virus-induced gene silencing (BSMV-VIGS) was employed to silence the *TaSWP73*, *TaSARD1*, and *TaICS1* genes in the Yannong 999 plants. For the BSMV-VIGS assay, about 200bp antisense (*as*) fragments of *TaSWP73*, *TaSARD1*, or *TaICS1* were amplified using the primers 5′AAGGAAGTTTAACACCAGCACCAGGGCCATC3′/5′AACCACCACCACCGTGATCCATGAGCATCGTAGG3′, 5′AAGGAAGTTTAATGGTTCTAGTATCTATAAG3′/5′AACCACCACCACCGTGTTTGGAACCAGTTATTCG3′, and 5′AAGGAAGTTTAATCAATGTCCCCATGTTTCC3′/5′AACCACCACCACCGTCTGTTGGTTGGTTTGGTGG3′. PCR products were cloned into the pCa-γbLIC vector through the ligation-independent cloning technique to create the *Agrobacterium*-mediated BSMV-VIGS constructs BSMV-*TaSWP73as*, BSMV-*TaSARD1as*, and BSMV-*TaICS1as.* The BSMV-VIGS assay silencing the indicated genes was performed as previously described [47]. Briefly, the construct DNA of pCaBS-α, pCaBS-β, and pCa-γbLIC derivatives (BSMV-*γ*, BSMV-*TaSWP73as*, BSMV-*TaSARD1as*, or BSMV-*TaICS1as*) was separately transformed into the *Agrobacterium tumefaciens* strain GV3101. Agrobacteria grown in LB liquid media were harvested and resuspended in infiltration buffer (10 mM MgCl_2_, 100 µM acetosyringone, and 10 mM MES). Equal amounts of cell suspension harboring pCaBS-α, pCaBS-β, and pCa-γbLIC derivatives were mixed for the agroinfiltration of *Nicotiana benthamiana* leaves. After maintenance in a growth chamber for 12 days post infiltration, the infiltrated *N. benthamiana* leaves were ground, and the sap was inoculated onto the two-leaf stages of wheat plants. The newly grown wheat leaves (n = 5) with virus symptoms about two weeks post BSMV infection were randomly collected for further experiments like gene expression analysis, wheat–*B.g. tritici* interaction characterization, and nucleosomal occupancy analysis. For the *B.g. tritici* microcolony formation analysis, the newly grown upper leaves with virus symptoms were collected and subjected to inoculation with *B.g. tritici* strain E09 conidia. About 72 h post *B.g. tritici* inoculation, leaf samples were fixed in an ethanol–acetic acid solution (1:1, *v*/*v*) and kept in a destaining solution (lactic acid–glycerol–water, 1:1:1, *v*/*v*/*v*). Thereafter, *B.g. tritici*-infected leaves were stained with 0.1% (*w*/*v*) Coomassie brilliant blue R250 to visualize the fungal epiphytic structure under a microscope. About 2000 *B.g. tritici*–wheat interaction sites (randomly chosen) were analyzed in one experiment. For the *B.g. tritici* microcolony index (MI%) analysis, three technical replicates using replicate samples were statistically analyzed, and the data are presented as the mean ± SE (Student’s *t*-test; ** *p* < 0.01). All the *B.g. tritici* microcolony index (MI%) analyses were repeated in three biological replicates using independently prepared samples with similar results.

### 4.4. Single-Cell Transient Gene Overexpression Assay and B.g. tritici Haustorium Formation Analysis

For the single-cell transient gene overexpression assay, the coding regions of *TaSWP73-2A*, *TaSWP73-2B*, and *TaSWP73-2D* were amplified using the primers 5′GGGGACAAGTTTGTACAAAAAAGCAGGCTTC ATGGCCACCGGTGGCAACC3′/5′GGGGACCACTTTGTACAAGAAAGCTGGGTCTCAAGAACCACCAGCACCA3′, 5′GGGGACAAGTTTGTACAAAAAAGCAGGCTTCATGGCCACCGGCGGCAACC3′/5′GGGGACCACTTTGTACAAGAAAGCTGGGTCTCAAGAACCACCAGCACCA3′, and 5′GGGGACAAGTTTGTACAAAAAAGCAGGCTTCATGGCCACCGGCGGCAACC3′/5′GGGGACCACTTTGTACAAGAAAGCTGGGTCTCAAGAACCACCAGCACCA3′. PCR products were cloned into pIPKb001, an expression vector driven by the maize ubiquitin promoter, to create the pIPKb001-*TaSWP73-2A* (for OE-*TaSWP73-2A*), pIPKb001-*TaSWP73-2B* (for OE-*TaSWP73-2B*), and pIPKb001-*TaSWP73-2D* (for OE-*TaSWP73-2D*) constructs using GATEWAY cloning technology (Invitrogen). The single-cell transient gene overexpression assay was performed as previously described [38]. The β-glucuronidase (GUS) reporter gene was co-delivered into the wheat epidermal cell to mark the transformed cells and better visualize the fungal haustorium in these cells. The pIPKb001 overexpression constructs were mixed with the GUS reporter vector at a 1:1 molar ratio before coating the DNA microcarrier. The exogenous DNA on the microcarrier was delivered into the wheat epidermal cell through a particle inflow gun (Bio-Rad, Hercules, CA, USA). The inoculation of *B.g. tritici* conidia spores was performed at least 16 h post bombardment. The leaf segments were stained for GUS activity 48 h post *B.g. tritici* spore inoculation and kept in a destaining solution. Before mounting for microscopy, the *B.g. tritici*-infected wheat leaves were stained with Coomassie blue to visualize the fungal epiphytic structure. About 50 *B.g. tritici*-infected wheat epidermal cells (randomly chosen) were analyzed in one experiment. For the *B.g. tritici* haustorium index (HI%) analysis, three technical replicates using replicate samples were statistically analyzed, and the data are presented as the mean ± SE (Student’s t-test; ** *p* < 0.01). All the *B.g. tritici* haustorium index (HI%) analyses were repeated in three biological replicates using independently prepared samples with similar results.

### 4.5. SA Measurement

Free SA was analyzed using High-Performance Liquid Chromatography (HPLC), as previously described [48]. Briefly, the newly grown wheat leaves (n = 5) with virus symptoms about two weeks post BSMV infection were randomly collected and ground with liquid nitrogen into powder and then homogenized in 70% ethanol (*v*/*v*) containing the internal standard ortho-anisic acid. After centrifugation, the supernatant was collected, and the pellet was homogenized with 90% *v*/*v* methanol. After centrifugation, both supernatants were pooled and evaporated under vacuum. Then, 5% trichloroacetic acid was added to the remaining aqueous solution. After centrifugation, the supernatant was collected and mixed with ethyl acetate/cyclohexane. After centrifugation, the upper organic phase was collected. For SA quantification, organic phases were resuspended in HPLC starting solvent (methanol 40%, water 60%, acetic acid 1%) and analyzed by a reverse-phase HPLC column. The free SA amount was calculated in ng mg^−1^ fresh weight (FW) with reference to the amount of internal standard. For the free SA measurement, three technical replicates using replicate samples were statistically analyzed, and the data are presented as the mean ± SE (Student’s *t*-test; ** *p* < 0.01). All the free SA measurements were repeated in three biological replicates using independently prepared samples with similar results.

### 4.6. Nucleosomal Occupancy Analysis

The nucleosome occupancy micrococcal nuclease (MNase) assay was conducted to analyze the chromatin assembly structure in the *TaSARD1* promoter regions as previously described [29]. Briefly, the newly grown wheat leaves (n = 5) with virus symptoms about two weeks post BSMV infection were randomly collected and cross-linked and then subjected to nuclear isolation and MNase digestion. Genomic DNA was then recovered and underwent qPCR analysis to analyze the *TaSARD1* promoter regions using the primers 5′CTGTGACTTCATGCTCAAG3′/5′CCAAATCATCTAACTTTCC3′, 5′ATGTACACTGAAATTAATC3′/5′GATGCAGGTAGAAAGCAGG3′, 5′TGAATTGTCAAATGTCTCT3′/5′GTTGGTAGCGTCTCTTATC3′, 5′TGGTGCGTGCACTGAAATC3′/5′AGCTGCAGGCAGCTAGGGA3′, 5′ACGGGCTGCCCTGACACTC3′/5′GAGCTCCTGAAGCAGCTGG3′, 5′CACCCGACATCAAAACAAC3′/5′GCCGTTAGTTTAGGACAGG3′, 5′GCTTTGCAAAGCAACTTGG3′/5′CTGGCGTAATGATAAGAAG3′, 5′ACAAATAACCATCGACCCA3′/5′ATTAGTTGTTTATTTAATT3′, 5′TACAAAGCGATGAATGCCA3′/5′TACTCTGTTGCTATGTTAG3′, and 5′AACCATCGACCACCTATTG3′/5′CAAGGCTTCGAGCTCCCAA3′. Nuclei without MNase digestion treatment were employed as the input control. For the MNase assay, three technical replicates using replicate samples were statistically analyzed, and the data are presented as the mean ± SE (Student’s *t*-test; ** *p* < 0.01). All MNase assays were repeated in three biological replicates using independently prepared samples with similar results.

### 4.7. Phylogenetic Tree Reconstruction

SWP73 and ICS1 homologs from *Arabidopsis*, *Brachypodium*, maize, rice, and wheat were subjected to protein alignment with Clustal W, and phylogenetic trees were reconstructed using the Neighbor-Joining method with 1000 bootstraps by MEGA 7 (Molecular Evolutionary Genetics Analysis) software.

### 4.8. Statistical Analysis

For the statistical analysis of gene expression, SA measurement, nucleosomal occupancy in gene promoter regions, *B.g. tritici* microcolony formation, and *B.g. tritici* haustorium formation, at least three independent experiments were performed for each assay, and at least 5 wheat leaves (for the RT-qPCR, SA measurement, nuclear run-on, and MNase assay), 50 *B.g. tritici*-infected wheat epidermal cells (for the *B.g. tritici* haustorium index (HI%) analysis), and 2000 *B.g. tritici*–wheat interaction sites (for the *B.g. tritici* microcolony index (MI%) analysis) were analyzed in one experiment or were randomly chosen for each group. Three technical replicates per assay were analyzed using Student’s *t*-test, and the value represents the mean ± standard deviation (*n. s. p* > 0.05, * 0.01 < *p* < 0.05, ** *p* < 0.01; *n. s.* represents no significant difference). These assays were repeated in three independent biological replicates using independently prepared samples with similar results.

## 5. Conclusions

Herein, we characterized the function of the wheat chromatin remodeling protein TaSWP73 in regulating the wheat–*B.g. tritici* interaction and demonstrated that TaSWP73 negatively regulates wheat post-penetration resistance against *B.g. tritici.* The overexpression of *TaSWP73* attenuates wheat post-penetration resistance against *B.g. tritici*, while the silencing of *TaSWP73* potentiates SA biosynthesis and activates post-penetration resistance against *B.g. tritici.* Furthermore, we found that chromatin in the promoter regions of *TaSARD1,* an activator gene of SA biosynthesis, is marked by high nucleosome occupancy in the *TaSWP73*-silenced wheat leaves. The silencing of *TaSARD1* could suppress SA biosynthesis and attenuate post-penetration resistance against *B.g. tritici* with a lack of *TaSWP73.* In addition, we identified TaICS1 as an essential component of wheat SA biosynthetic machinery and found that potentiated SA biosynthesis and increased post-penetration resistance against *B.g. tritici* with a lack of *TaSWP73* could be suppressed by the silencing of *TaICS1* expression. These results collectively suggest that the wheat chromatin remodeling protein *TaSWP73* negatively regulates post-penetration resistance against *B.g. tritici* probably via the suppression of the TaSARD1-TaICS1-SA pathway. These findings shed novel light on the epigenetic mechanism underlying wheat–*B.g. tritici* interactions, and genetically manipulating the *TaSWP73*, *TaSARD1*, and *TaICS1* genes characterized in this study might provide a promising new avenue to improve wheat post-penetration resistance against powdery mildew disease in future research.

## Data Availability

Data presented here are available on request through correspondence.

## Supplementary Materials

The supporting information can be downloaded at: https://www.mdpi.com/article/10.3390/ijms26062590/s1.
