# Supplementary material for: Wheat Chromatin Remodeling Protein TaSWP73 Contributes to Compatible Wheat–Powdery Mildew Interaction
Source: Int J Mol Sci. 2025 Mar 13;26(6):2590. doi: 10.3390/ijms26062590 (PMC11942214; doi:10.3390/ijms26062590)
Supplement: Supplementary file 1 [file ijms-26-02590-s001.zip › ijms-3459848-supplementary.pdf]

Supplemental Data

Wheat Chromatin Remodeling Protein TaSWP73 Contributes to the Compatible Wheat-Powdery Mildew Interaction

Yixian Fu[1,#], Zige Yang[1,#], Jiao Liu[1], Xiaoyu Wang [1], Haoyu Li [1], Pengfei Zhi [1], and Cheng Chang [1,*]

College of Life Sciences, Qingdao University, Qingdao 266071, China;

# These authors contributed equally to this work

* Correspondence: cc@qdu.edu.cn

Table of Contents:

Figure S1. Alignment of nucleotide sequences at the coding regions of allelic *TaSWP73-2A*, *TaSWP73-2B*, and *TaSWP73-2D*.

Figure S2. RT-qPCR analysis of *TaSWP73* expression levels in response to *B.g. tritici* inoculation.

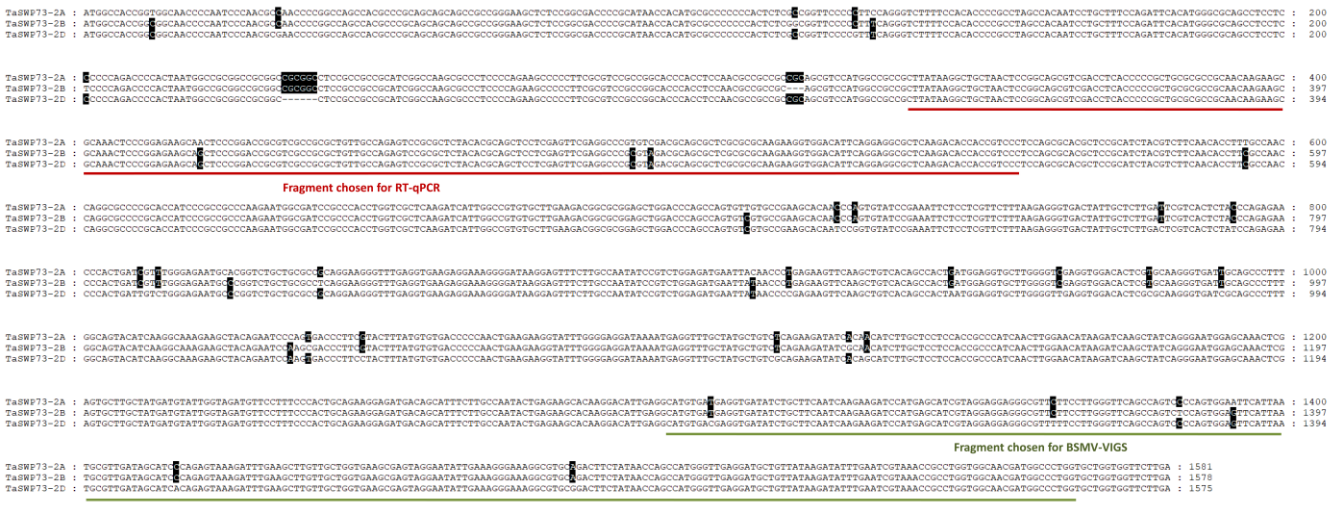

Figure S1. Alignment of nucleotide sequences at the coding regions of allelic *TaSWP73-2A, TaSWP73-2B,* and *TaSWP73-2D*. The nucleotide sequences of *TaSWP73-2A*, *TaSWP73-2B*, and *TaSWP73-2D* are derived from wheat A, B and D genome, respectively. Variations among *TaSWP73-2A*, *TaSWP73-2B*, and *TaSWP73-2D* nucleotide sequences are shaded in black. Regions chosen for the *TaSWP73* expression analysis and gene silencing are underlined.

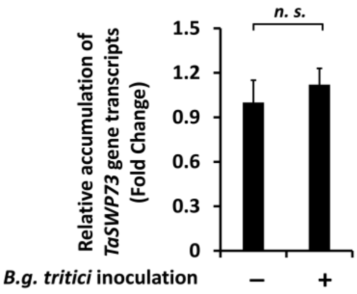

Figure S2 RT-qPCR analysis of *TaSWP73* gene transcript accumulation levels in response to *B.g. tritici* inoculation. Three technical replicates per treatment were statistically analyzed and data are presented as the mean±SE (Student's t-test; ** P<0.01), and these assays were repeated in three independent biological replicates with similar results.
